# A Promising Role of TGF-β Pathway in Response to Regorafenib in Metastatic Colorectal Cancer: A Case Report

**DOI:** 10.3390/medicina57111241

**Published:** 2021-11-13

**Authors:** Simona De Summa, Katia Danza, Brunella Pilato, Giuseppina Matera, Rossella Fasano, Angela Calabrese, Rosanna Lacalamita, Nicola Silvestris, Stefania Tommasi, Antonella Argentiero, Oronzo Brunetti

**Affiliations:** 1 Molecular Diagnostics and Pharmacogenetics Unit, IRCCS Istituto Tumori “Giovanni Paolo II”, 70124 Bari, Italy; s.desumma@oncologico.bari.it (S.D.S.); danzakatia@gmail.com (K.D.); brunellapilato@gmail.com (B.P.); g.matera92@gmail.com (G.M.); rosanna.lacalamita@gmail.com (R.L.); s.tommasi@oncologico.bari.it (S.T.); 2 Medical Oncology Unit, IRCCS Istituto Tumori “Giovanni Paolo II” of Bari, 70124 Bari, Italy; rossella.fasano.93@gmail.com (R.F.); n.silvestris@oncologico.bari.it (N.S.); argentieroantonella@gmail.com (A.A.); 3 Radiology Unit, IRCCS Istituto Tumori “Giovanni Paolo II”, 70124 Bari, Italy; acalabrese22@gmail.com; 4 Department of Biomedical Sciences and Human Oncology, University of Bari “Aldo Moro”, 70124 Bari, Italy

**Keywords:** colorectal cancer, TGF-b, regorafenib

## Abstract

Colorectal cancer (CRC) is one of the most common cancer types around the world. The prognosis of patients with advanced diseases is still poor in spite of currently available therapeutic options. Regorafenib is an oral tyrosine kinase inhibitor (TKI) approved to treat refractory metastatic colorectal cancer (mCRC). We investigated Somatic mutations in several genes involved in immunological response and cancer progression in both long/short responder mCRC patients who underwent third-line therapy with regorafenib to identify predictive biomarkers of response using Ion Torrent PGM sequencing and bioinformatic tools. We found Somatic mutations in TGFBR1, TGFBR2, and TGFBR3 genes in primary tumor and metastases samples of long-responder patients. Furthermore, our bioinformatic results show that they were mainly enriched in immune response, cell junction, and cell adhesion in long responder patients, particularly in primary tumor and metastatic sites. These data suggest that the TGF-b pattern could be the leading actor of a prolonged response to this drug.

## 1. Introduction

Colorectal cancer (CRC) is the third leading cause of cancer death globally, and its incidence is steadily rising in developing nations [1,2,3]. In recent years, many targeted therapeutic strategies have been proposed for metastatic colorectal cancer (mCRC) patients [4]. Regorafenib is an oral type II multi-kinase inhibitor that inhibits the activity of vascular endothelial growth factor receptor 1, 2, 3 (VEGFR-1, -2, -3), platelet-derived growth factor receptors, fibroblast growth factor receptors (FGFR), tyrosine kinase receptor with immunoglobulin-like and EGF-like domains 2 (TIE-2), and oncogenic receptor tyrosine kinases [5], showing an impact on angiogenesis and metastasis processes. Observational post-marketing studies have confirmed its efficacy in the face of a non-negligible toxicity profile [6,7]. It has been approved in the third or later lines of treatment for patients with chemorefractory mCRC, according to the results of two randomized phases III trials (CORRECT and CONCUR) [8,9]. Unlike anti-EGFR monoclonal antibodies for which well-defined molecular predictive factors are available [10], results for anti-angiogenic drugs are still inconclusive [11]. However, since its introduction into the clinical setting, single exceptional responders to this drug have been reported [12,13,14,15]. Thus far, identifying useful predictive factors to identify the best candidates for such a therapy represents a relevant clinical challenge. 

A more remarkable progression-free survival (PFS) benefit for regorafenib has been observed in patients showing epithelial-mesenchymal transition (EMT) phenotype and higher TGF-β pathway activation [16]. TGF-β pathway promotes angiogenesis and EMT and inhibits the growth of epithelial and immune cells [17,18]. Loss of SMAD4, a tumor suppressor gene, disrupts R-SMAD-SMAD4 complexes in the canonical TGF-β signaling, leading to the deregulation of several SMAD4-related target genes, such as VEGF-A, VEGF-C, and β-catenin [19]. A strict and mutual regulation between vasculature normalization and immune activation has been described in the tumor microenvironment [20]. Consequently, in this study, we evaluate somatic mutations of several genes involved in immunological response and cancer progression, with the aim to identify, through a NGS platform, potential biomarkers of response to regorafenib in one very long and short responder mCRC patient. 

## 2. Materials and Methods

### 2.1. Patients’ Characteristics

The clinical history of the 58-year-old mCRC who presented a prolonged progression-free survival (PFS) (16 months) to third-line regorafenib has been previously published [21]. 

The control case was a 54-year-old female mCRC patient who received the third-line treatment with regorafenib with a PFS of 4 months. This study was approved by the Local Ethical Committee (Prot. N.709/CE). Both patients provided informed consent.

### 2.2. Sample Processing

All surgical samples were formalin-fixed and paraffin-embedded (FFPE). Tumor sections were cut from each FFPE block: one section was stained by hematoxylin/eosin to confirm and locate the tumor, and consecutive sections were used for immunohistochemistry and gene expression analyses. 

#### 2.2.1. DNA and RNA Extraction

Three to six FFPE tissue sections (6 μm thick) with adequate tumor cellularity, selected by a pathologist (>50%), were macro dissected and subjected to the QIAamp DNA FFPE Tissue Kit (Qiagen, Venlo, The Netherlands) for DNA isolation and the RNeasy FFPE Kit (Qiagen) for RNA isolation, according to the manufacturer’s protocols. DNA was also isolated from blood samples using the QIAamp DNA Blood Midi Kit (Qiagen). DNA and RNA concentrations were measured using the Qubit 2.0 fluorometer (Thermo Fisher Scientific, Waltham, MA, USA).

#### 2.2.2. Ion Torrent PGM Sequencing

The sequencing which has been used in the current study has been reported in our previous study [22]. Briefly, two custom panels have been designed through the Ion Ampliseq designer tool, one including the coding region of 41 genes to detect Somatic mutations and one to study the gene expression of 95 genes. Both include genes involved in immune regulation and inflammation. Variant calling and filtering have been described in [22]. In particular, somatic variants were called when matching the following conditions: DP > 50, VD > 20, and QUAL > 30. Furthermore, the Cancer hotspot Panel V2 has been used to identify druggable alterations. Briefly, the call set has been generated merging results from the Somatic High-Stringency Variant Caller plugin of the Torrent Suite and the Vardict [23] algorithm. Germline variants were filtered out using a pool of healthy controls. Annovar [24] was used to annotate variants functionally. The Oncoprint plot has been designed with the ComplexHeatmap R package [25].

#### 2.2.3. MSI Analysis

MSI Analysis was performed by using Real-Time PCR (Easy PGX Diatech) to detect the microsatellite region instability in tumor samples. The analysis of 8 mononucleotide markers (BAT-25, BAT-26, NR-21, NR-22, NR-24, NR-27, CAT-25, and MONO-27) was based on the denaturation profile and compared to a positive control with a stable profile and followed manufacture instructions (EasyPGX^®^ ready MSI cat.no. RT033).

The EasyPGX^®^ Analysis Software was used to analyze all the melting temperatures and to generate a melting profile from each sample and the positive control. The software automatically calculates the status of global instability (MSS, MSI-L, MSI-H) from the number of unstable markers. 

A tumor with high instability (H-MSI) has ≥2 unstable markers, and a tumor with L-MSI (low instability) has 1 unstable marker. For all L-MSI tumors, it is needed to repeat the analysis comparing the melting profile of the tumor tissue to the melting profile of normal tissue to exclude possible germline instability.

### 2.3. Protein-Protein Interaction (PPI) Network and Pathway Enrichment Analysis

Metascape (Available online: https://metascape.org/gp/index.html#/main/step1 (accessed on 10 July 2020)), an online resource, has been used to depict biological networks, including the interaction between mutated genes and to perform functional enrichments.

## 3. Results

### 3.1. Mutational Pattern

**Figure 1:**
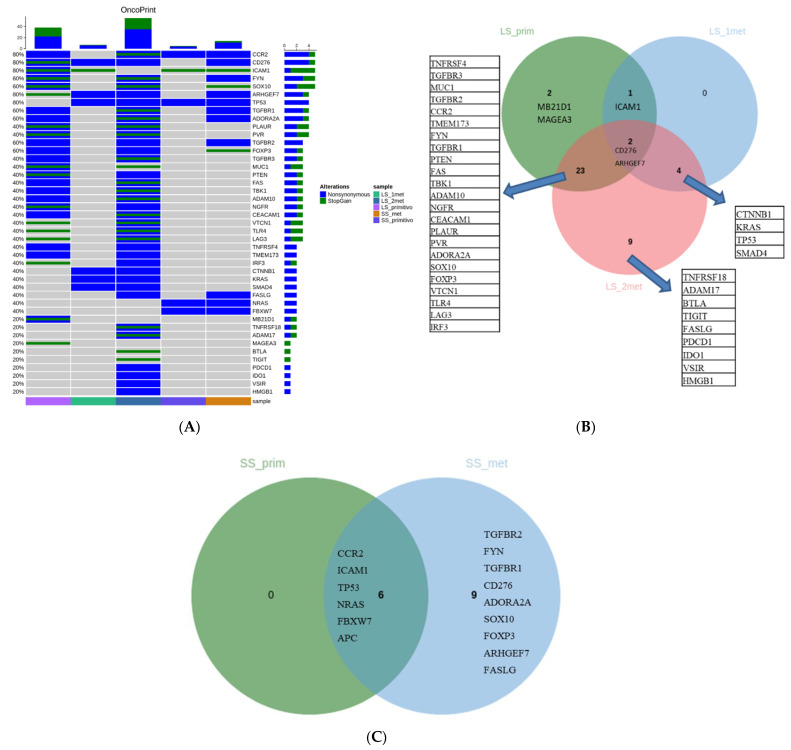
(**A**) Oncoprint including pathogenic alterations detected in the five analyzed samples. (**B**) Venn diagram of the alterations detected in the long survival samples (primary CRC and the two metastatic samples). (**C**) Venn diagram of the alterations detected in the short survival samples (primary CR Cand metastatic samples). LS_prim: primary tumor of the long survival case; LS_1met, LS_2met: metastatic samples of the long survival case; SS_prim: primary tumor of the short survival case; SS_met: metastatic sample of the short survival patient.

Through a custom targeted NGS panel including 41 genes, long-responder and short-responder samples were sequenced. In detail, long-responder samples included a primary tumor and two ovarian metachronous metastases. The control case included a primary tumor and one lung metastasis. The primary tumor and the first metastasis showed a distinct pattern of alterations (only CD276, ICAM1, and ARHGEF7 mutations are common) (Figure 1A). The second metastasis shared almost all mutations detected in the primary tumor: four alterations were detected in the first metastasis with 9 private mutations (Figure 1B). The mutational pattern of the short-responder in the primary and metastatic samples reflects a simple mechanism of clonal evolution (Figure 1C). Indeed, we observed that metastasis has the same alterations as the primary tumor samples with a further 9 private mutations. Microsatellite status has been checked in all samples, which were found to be stable (MSS).

### 3.2. Functional Enrichment and PPI Network

**Figure 2:**
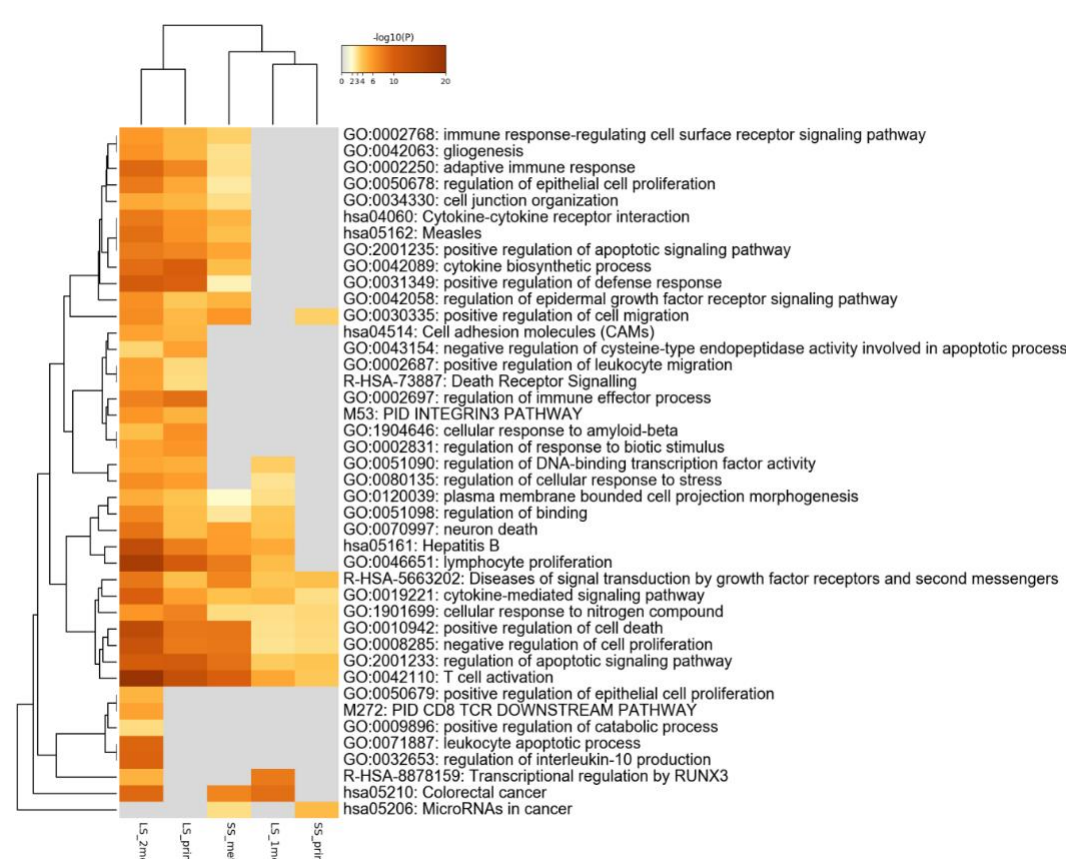
Global pathway enrichment of the detected alterations. LS_prim: primary tumor of the long survival case; LS_1met, LS_2met: metastatic samples of the long survival case; SS_prim: primary tumor of the short survival case; SS_met: metastatic sample of the short survival patient.

The global pathway enrichment is displayed as a heatmap (Figure 2). Terms related to immune response, cell junction, and cell adhesion molecules are the most enriched in long-responder patients, particularly in the primary tumor and second metastasis samples. 

**Figure 3:**
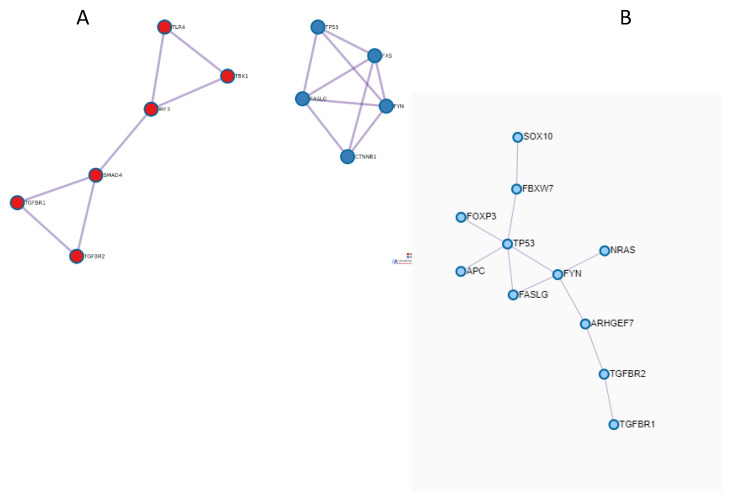
Interaction networks of the alterations detected in (**A**) long survival samples and (**B**) in short survival samples. In Table 1, significantly enriched terms are shown according to the sub-network color scale. PPI network was built up also for the short responder case (Figure 3B) and functionally enriched (Table 1).

The PPI network, including proteins with relative genes which were found to be altered in the second metastatic samples, was built up. It can be observed that two sub-networks were identified, which were, in turn, functionally enriched (Figure 3A). 

## 4. Discussion

Despite improvements in the management of mCRC, drug resistance remains a clinical challenge in the advanced stage. Regorafenib was the first approved multikinase inhibitor with survival benefits in unselected mCRC patients who had exhausted current standard therapies [8,9]. Regorafenib inhibits the activity of several protein kinases active in the regulation of angiogenesis, oncogenesis, and in the modulation of the tumor microenvironment. Although several clinical and biological parameters have been investigated, there are no useful predictive markers for regorafenib treatment [26,27]. In this study, we explored Somatic mutations of genes involved in immunological and inflammation response in one very long-responder and one short-responder mCRC patient to regorafenib. Recently, an immune profile that correlates with the outcome in mCRC patients treated with regorafenib has been reported, suggesting a cytokine signature able to discriminate patients who might derive a benefit from regorafenib treatment [28]. In particular, the plasma basal level of proteins TNF-α and TGF-β before treatment might be useful to identify mCRC patients that do not benefit from regorafenib and show the progression of the disease. According to these results, our data show most mutated genes involved in TGF-β signaling in long-responder mCRC patients to regorafenib therapy.

In particular, Somatic mutations in TGFBR1, TGFBR2, and TGFBR3 genes were found in the primary tumor and metastatic samples of our long-responder patient. TGF- β was identified as a major signaling pathway in CRC invasion and metastasis. Its activation generally promotes CRC invasion and metastasis through EMT, whereas it suppresses cancer immunity in the tumor microenvironment [29]. The relevance of TGF-β signaling in the acquisition of an invasive phenotype was also demonstrated by our PPI subnetwork obtained by protein products of altered genes found in the last metastasis of the long-responder mCRC patient. This subnetwork was enriched by terms related to “Signaling by TGF-beta Receptor Complex in Cancer” and “TGF-beta receptor signaling activates SMADs”. No similar terms were observed by the enrichment of the PPI network highlighted by protein products of altered genes found in metastases of the short-responder.

Interestingly, in a study, researchers showed a deleterious mutation in the SMAD4 gene in the long-responder metastatic patient [30]. Martinelli et al. [16] reported a greater PFS benefit for regorafenib therapy in patients with SMAD4 gene mutation characterized by the activation of TGFβ signaling and upregulation of an EMT pathway [31]. Authors observed mutation in SMAD4 in two long-responder patients, suggesting a key role of this gene in regorafenib response [16]. Down-regulation or mutation of SMAD4 underlies a more rapid protein degradation, leading to pancreatic cancer cell cycle arrest and apoptosis [32]. A key role of this member of TGFβ signaling has also been reported in CRC cells, in which deletion of SMAD4 decreased the number of TAMs in the tumor microenvironment, contributing to unfavorable prognoses [19].

Regorafenib appears to participate in the immune system with tumor interaction in different ways, including inhibition of tyrosine kinase receptor CSF1R, which is involved in macrophage proliferation [33]. Recently, a strict and mutual regulation between vasculature normalization and immune activation has been described in the tumor microenvironment [20]. In conclusion, we can hypothesize that the TGF-b pattern could be the leading actor of a longer response; however, all our results should be better explored in a larger cohort.

## Figures and Tables

**Table 1:** The significant genes enriched in the different pathways. In red are the enriched pathways for the network in Figure 3A; in blue is the sub-network in Figure 3B.

Category	GO	Description	LogP
KEGG Pathway	hsa05161	Hepatitis B	−10
Reactome Gene Sets	R-HSA-3304349	Loss of Function of SMAD2/3 in Cancer	−9.5
Reactome Gene Sets	R-HSA-3304351	Signaling by TGF-beta Receptor Complex in Cancer	−9.3
GO Biological Processes	GO:0045351	type I interferon biosynthetic process	−8.9
Reactome Gene Sets	R-HSA-936964	Activation of IRF3/IRF7 mediated by TBK1/IKK epsilon	−8.2
GO Biological Processes	GO:0003198	epithelial to mesenchymal transition involved in endocardial cushion formation	−8
GO Biological Processes	GO:0032727	positive regulation of interferon-alpha production	−7.8
Canonical Pathways	M185	PID ALK1 PATHWAY	−7.7
GO Biological Processes	GO:0003272	endocardial cushion formation	−7.6
GO Biological Processes	GO:0035666	TRIF-dependent toll-like receptor signaling pathway	−7.5
GO Biological Processes	GO:0032647	regulation of interferon-alpha production	−7.5
GO Biological Processes	GO:0032728	positive regulation of interferon-beta production	−7.4
GO Biological Processes	GO:0032607	interferon-alpha production	−7.4
Reactome Gene Sets	R-HSA-2173789	TGF-beta receptor signaling activates SMADs	−7.4
GO Biological Processes	GO:0002756	MyD88-independent toll-like receptor signaling pathway	−7.3
GO Biological Processes	GO:0060317	cardiac epithelial to mesenchymal transition	−7.2
GO Biological Processes	GO:0003203	endocardial cushion morphogenesis	−7.2
GO Biological Processes	GO:2000826	regulation of heart morphogenesis	−7
GO Biological Processes	GO:0060412	ventricular septum morphogenesis	−6.9
GO Biological Processes	GO:0003197	endocardial cushion development	−6.9
GO Biological Processes	GO:1901216	positive regulation of neuron death	−8.9
GO Biological Processes	GO:2001233	regulation of apoptotic signaling pathway	−8.9
KEGG Pathway	hsa05162	Measles	−8.4
GO Biological Processes	GO:0097190	apoptotic signaling pathway	−8
Reactome Gene Sets	R-HSA-109581	Apoptosis	−7.8
Reactome Gene Sets	R-HSA-5357801	Programmed Cell Death	−7.8
KEGG Pathway	hsa05205	Proteoglycans in cancer	−7.6
GO Biological Processes	GO:0010942	positive regulation of cell death	−7.6
GO Biological Processes	GO:0043523	regulation of neuron apoptotic process	−7.5
GO Biological Processes	GO:2001234	negative regulation of apoptotic signaling pathway	−7.4
GO Biological Processes	GO:0051402	neuron apoptotic process	−7.3
GO Biological Processes	GO:0070266	necroptotic process	−7.3
GO Biological Processes	GO:0097300	programmed necrotic cell death	−7.1
GO Biological Processes	GO:0043525	positive regulation of neuron apoptotic process	−6.9
GO Biological Processes	GO:1901214	regulation of neuron death	−6.9
GO Biological Processes	GO:0070265	necrotic cell death	−6.8
GO Biological Processes	GO:0070997	neuron death	−6.7
KEGG Pathway	hsa01524	Platinum drug resistance	−6.6
KEGG Pathway	hsa05200	Pathways in cancer	−6.5
GO Biological Processes	GO:2001237	negative regulation of extrinsic apoptotic signaling pathway	−6.1

## Data Availability

Not available.
